# Steinmann pin augmentation versus locking plate constructs

**DOI:** 10.1007/s10195-016-0394-y

**Published:** 2016-02-16

**Authors:** Jeremy Ruskin, Paolo Caravaggi, Kathleen S. Beebe, Sondra Corgan, Linda Chen, Richard S. Yoon, Francis R. Patterson, John S. Hwang

**Affiliations:** 1 Department of Orthopaedic Surgery, Rutgers, The State University of New Jersey, New Jersey Medical School, Newark, NJ 07103 USA; 2 Movement Analysis Laboratory, Istituto Ortopedico Rizzoli, Via di Barbiano 1/10, 40136 Bologna, Italy; 3 Department of Orthopaedic Surgery, NYU Hospital of Joint Diseases, New York, NY USA

**Keywords:** Giant cell tumor, Steinmann pin, Locking plate, Oncology

## Abstract

**Background:**

Aggressive bone neoplasms, such as giant cell tumors, often affect the proximal tibia warranting bony resection via curettage leaving behind massive defects that require extensive reconstruction. Reconstruction is usually accomplished with poly(methyl methacrylate) (PMMA) packing supplemented with an internal fixation construct. The purpose of this study is to compare Steinmann pin augmentation to locking plate constructs to determine which offers the stiffer reconstruction option.

**Materials and methods:**

Large defects were created below the lateral condyle of fresh frozen tibias. The defects extended for an average of 35 mm beneath the lateral plateau in the frontal plane, and from the anterior to posterior cortex in the sagittal plane. Distally the defect extended for an average of 35 mm to the metadiaphyseal junction. In the Pin group, the tibias were reconstructed with three 4-mm diameter Steinmann pins placed in the medullary canal and PMMA packing. In the Plate group, the tibias were reconstructed with a 6-hole 3.5-mm LCP Proximal locking plate fixed to the proximal−lateral tibia utilizing seven 3.5-mm screws and PMMA packing. The tibias were tested for stiffness on a MTS machine by applying up to 400 N to the tibial plateau in force control at 5 N/s. Fatigue properties were tested by applying a haversine loading waveform between 200 N and 1,200 N at 3 Hz simulating walking upstairs/downstairs.

**Results:**

Locking plate constructs (801.8 ± 78 N/mm) had greater (*p* = 0.041) stiffness than tibial constructs fixed with Steinmann pins (646.5 ± 206.3 N/mm).

**Conclusions:**

Permanent deformation was similar between the Pin and Plate group; however, two tibia from the Pin group exhibited displacements >5 mm which we considered failure.

**Level of evidence:**

n/a.

## Introduction

Aggressive bone neoplasms, such as giant cell tumors, often affect the proximal tibia warranting bony resection via curettage that leaves behind massive defects that require extensive, stable reconstruction in order to maximize function [1]. Accomplishing stable, functional reconstruction in the proximal tibia with maintenance of long-term stability can be a challenging task. Typically, such defects are packed with poly(methyl methacrylate) (PMMA) cement. PMMA improves stability and allows ease of access if a second intervention if necessary. Given the large size of the defect created by excision of a giant cell tumor, PMMA packing is generally supplemented with an internal fixation device. Several past biomechanical studies have compared a variety of internal fixation constructs with regard to strength and durability in an effort to determine the best possible option to maximize clinical outcome [2–4]. In particular, Steinmann pin augmentation with PMMA exhibited superior strength and durability to cement alone when tested at physiological cycling and load-to-failure conditions [5]. Similarly, locking plate constructs with PMMA exhibited superior results when compared to pins with PMMA [6]. However, this locking plate construct study was performed in the distal femur, where load distributions during normal physiological or biomechanical activity exhibit different, less forceful load distributions when compared to the proximal tibia [7, 8].

To date, comparison between Steinmann pin augmentation constructs and locking plate constructs in the setting of proximal tibial defects has yet to be performed. The aim of this study is to compare the two constructs in order to determine which offers the stiffer, more durable reconstruction option.

## Materials and methods

### Specimen preparation

Thirty fresh-frozen tibias from human cadavers (average age at time of death 48.3 years; 6 female, 20 male, 4 sex unknown) obtained from the Musculoskeletal Transplant Foundation (Edison, NJ, USA) were used in this study. The tibias, double-bagged and conserved at –20 °C, were thawed at room temperature for at least 12 h and stripped of all soft tissues before testing. A high-speed burr (Medtronics, Fort Worth, TX, USA) was used to create a defect underneath the lateral plateau simulating the cavitary defect resulting from surgical removal via curettage of a giant cell tumor of the bone in the proximal tibia. Defects were created proportionally in each tibia. The defect extended for an average of 35 mm beneath the lateral plateau in the frontal plane and from the anterior to posterior cortex in the sagittal plane. Distally the defect extended for an average of 35 mm to the metadiaphyseal junction. Each tibia was cut at mid-diaphysis using a sagittal saw and cemented with PMMA in the center of a 3-inch high piece of PVC pipe, leaving an average of 13.3 cm exposed from cement to lateral plateau.

Twelve matched pairs of tibia were used in two experimental groups, and two other pairs along with two unpaired tibias were used as controls. The tibias from each experimental pair were randomly assigned to two different groups, ensuring that an equal number of left and right tibias were in each group to account for any differences in laterality. The groups were randomly assigned to two different reconstruction methods.

### Reconstruction techniques

**Fig. 1:**
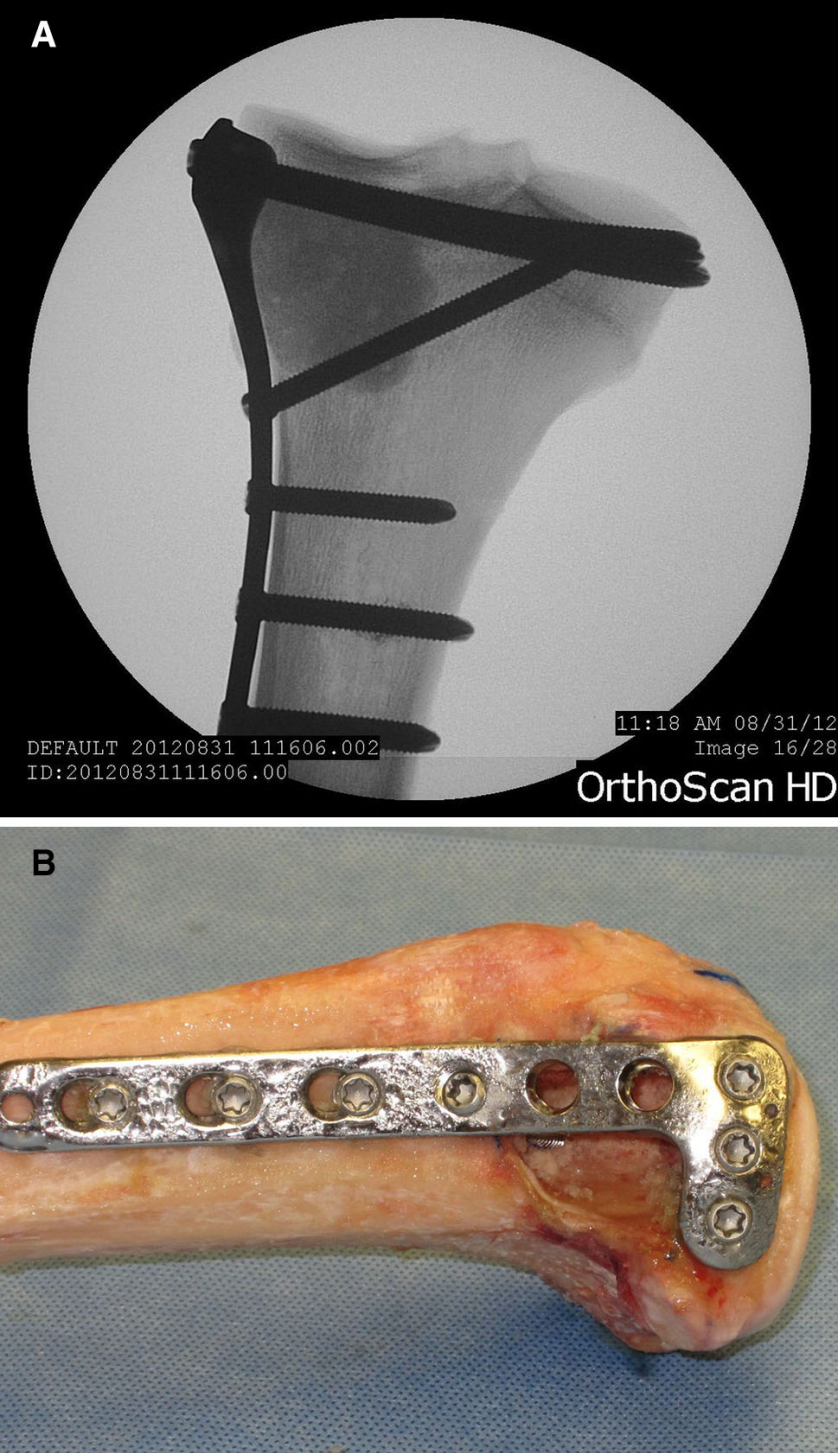
Radiograph after PMMA (**a**) and gross image before PMMA (**b**) of reconstruction with a locking plate

For the tibias in the Plate group, a 6-hole 3.5-mm LCP Proximal locking plate (Synthes, Paoli, PA, USA) was fixed to the proximal–lateral tibia following the company guidelines utilizing seven 3.5-mm screws (Fig. 1). The length of each screw was selected to ensure bicortical fixation.

**Fig. 2:**
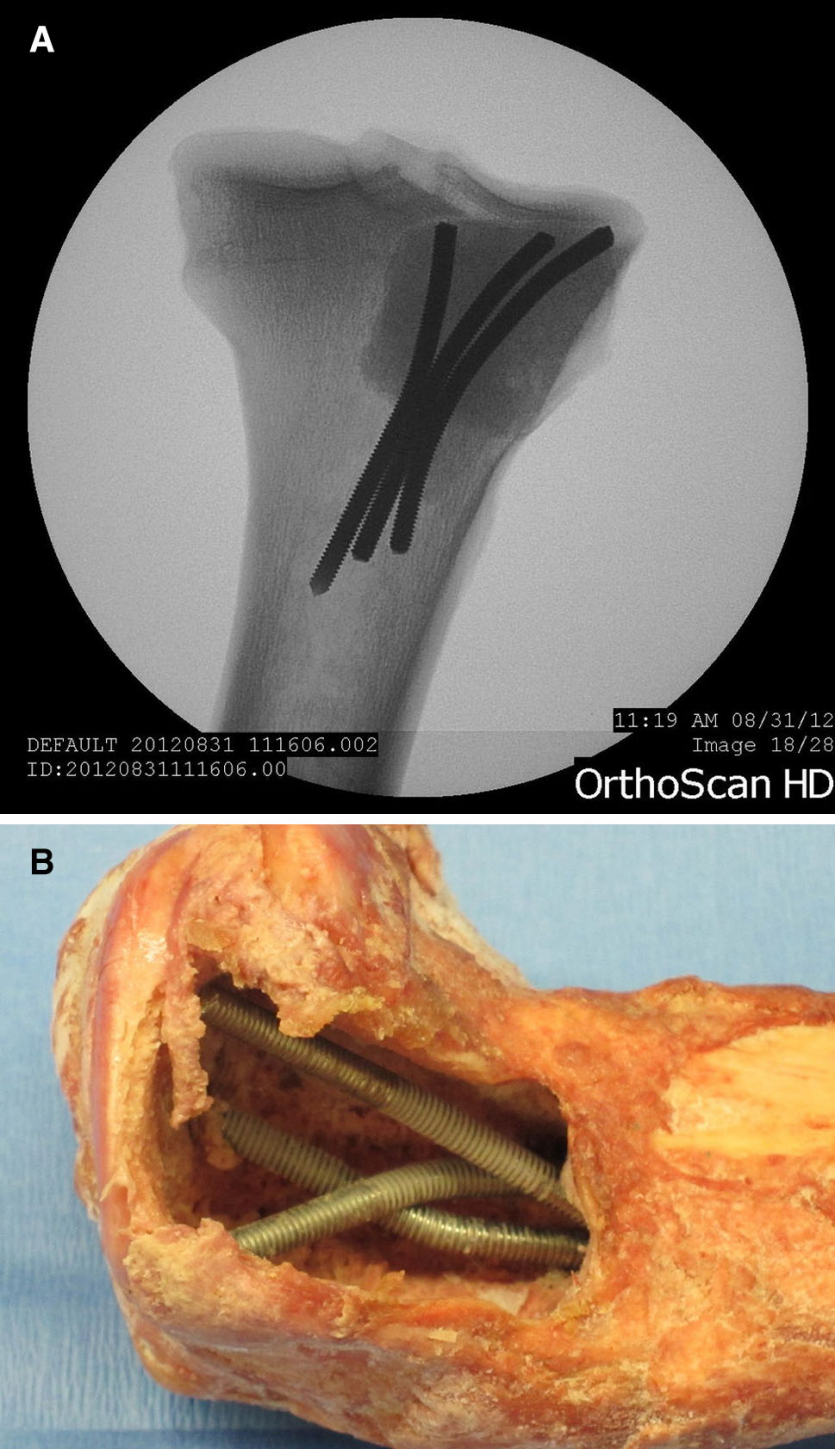
Radiograph after PMMA (**a**) and gross image before PMMA (**b**) of reconstruction with Steinmann pins

In the Pin group, three 4-mm diameter threaded Steinmann pins (Zimmer Inc., Warsaw, IN, USA) were inserted manually in the cavity and placed in the medullary canal distally. The pins were arranged in an ‘inverse tripod’ configuration so as to provide support to the tibial plateau on the proximal side (Fig. 2).

Following reconstruction, PMMA cement was molded into the defect, around the pins or locking plate and screws, and shaped to recreate the physiological contour of the lateral tibia. The PMMA was then allowed to cure.

### Testing

**Fig. 3:**
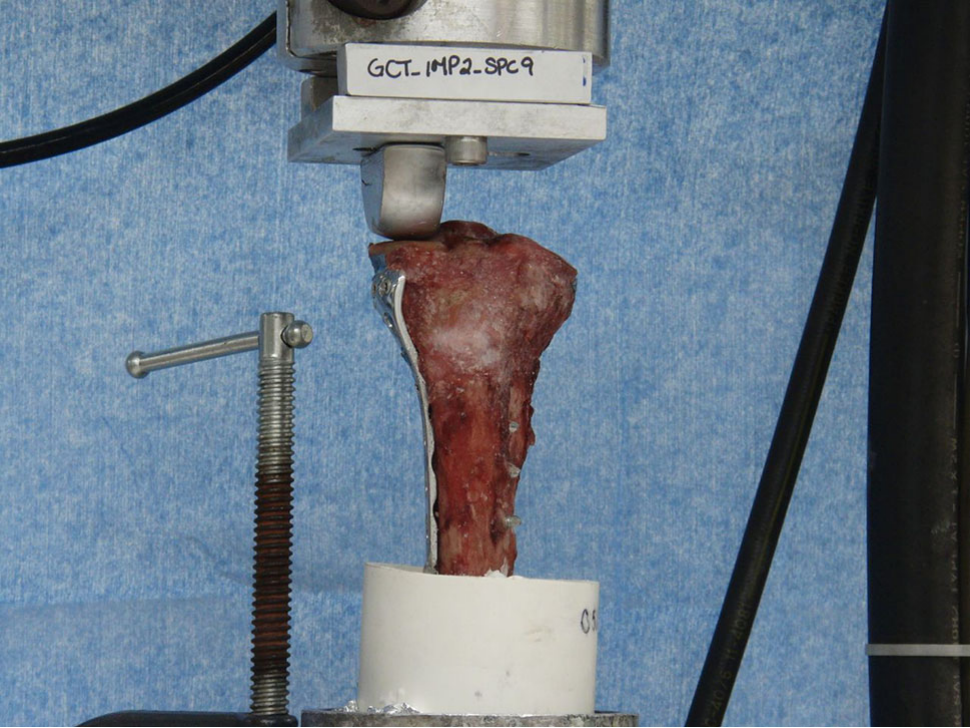
Plated specimen undergoing compression testing in MTS machine

Each tibia, set in its PVC fixture, was fixed to the loading frame of a MTS servohydraulic testing machine (MTS Corporation, Minneapolis, MN, USA) (Fig. 3). A custom unicondylar femoral component with a radius of 37 mm was fixed to the actuator arm of the MTS machine. The curvature and general dimensions of the condyle were consistent with those in the normal knee [9]. The femoral condyle was used for axial loading on the lateral tibial plateau, i.e., the side where the reconstruction had been performed. The actuator applied up to 400 N to the tibial plateau in force control at 5 N/s. Axial stiffness of the construct was determined from the slope of the force/displacement curve. Consequently, the fatigue properties of the construct were determined by application of a haversine loading waveform between 200 and 1,200 N at 3 Hz. The latter simulated a walking upstairs/downstairs loading pattern [10]. Permanent deformation after 20,000 cycles was recorded. Displacement was measured directly by the MTS machine as the vertical change in position of the lateral tibial plateau upon loading. Any visible sign of cracks or bone deformations were recorded at the end of each cyclic loading. The specimen was assumed to fail for displacements >5 mm.

### Statistical analysis

Differences in the biomechanical properties between the two groups were assessed with one-way Anova and Mann–Whitney tests. A *p* value <0.05 was considered significant.

## Results

**Fig. 4:**
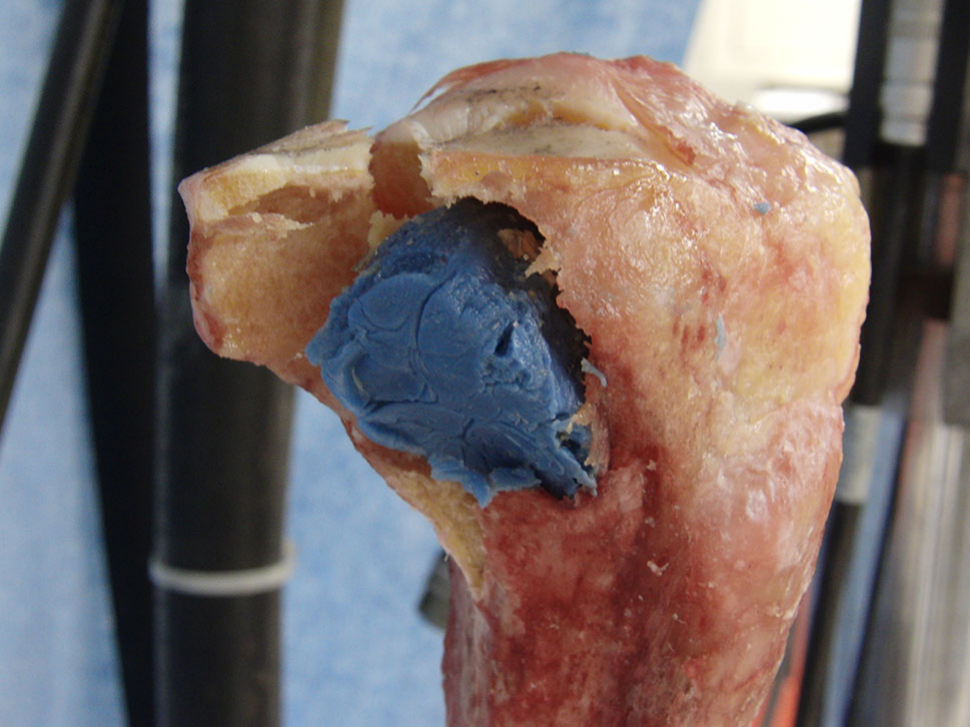
Tibia reconstructed with Steinmann pins that failed. Note the fracture line extending posteromedially from the lateral edge of the plateau. There is also a secondary fracture line extending from the lateral tibial spine to the midportion of the primary fracture line in a ‘T’ configuration

Consistency was found in the biomechanical properties within each implant group. The axial stiffness of the Plate group (801.8 ± 78 N/mm) was similar to that of the control group but significantly larger than that measured in the Pin group (646.5 ± 206.3 N/mm, *p* = 0.041; Table 1). All but two specimens from the Pin group (Fig. 4), and all specimens of the Plate group, sustained the cyclic test without failing. The two tibias that failed in the Pin group underwent significant damage with cracks of the plateau and pin displacement >5 mm. In both the Pin and Plate group, for those specimens that did not fail, permanent deformations following the cyclic test were slightly larger than 1 mm (Table 1). No statistical difference was found in the permanent deformation at 200 and 1,200 N after 20,000 cycles between the two groups.

**Table 1:** Mean (±SD) of the biomechanical parameters recorded in the study

	Control	Plate group	Pin group
Axial stiffness (N/mm)	799.2 (17.2)	801.8 (78.0)*	646.5 (206.3)
Permanent deformation after 20,000 cycles at 1,200 N (mm)	0.52 (0.02)	1.16 (0.33)	1.55 (0.59)
Permanent deformation after 20,000 cycles at 200 N (mm)	0.40 (0.09)	1.04 (0.33)	1.40 (0.56)

Mean permanent deformations following cyclic test were calculated across 12 specimens for the Plate group and 10 specimens for the Pin group, since two specimens from this group failed during the test

* One-way Anova statistically different at *p* < 0.05

## Discussion

Given the large bony cavity that results from giant cell resection via curettage, reconstruction of the cavity is necessary. Prior studies have demonstrated the importance of using PMMA and Steinmann pins to reconstruct these defects [5, 11–13, 15]. The advent of locking plate technology allows for a newer method of reconstructing these cavities [14]. Only one other published study looks at using locking plates to reconstruct giant cell tumor defects after resection, and this was performed for the distal femur [6]. Therefore, the goal of this study was to evaluate the effect of using proximal tibia locking plates versus Steinmann pin augmentation on cemented proximal tibia defects.

In this study, large defects simulating those left behind after curettage of a giant cell tumor were created in the lateral proximal tibia of human cadaver tibias. The defects were fixed with either three Steinmann pins in an inverted tripod configuration or a proximal tibia locking plate. The reconstructed tibias were subjected to loads to determine their stiffness and fatigue properties. As expected from our hypothesis, the tibias reconstructed with locking plates showed a higher stiffness than those reconstructed with Steinmann pins (801.8 ± 78 N/mm vs 646.5 ± 206.3 N/mm, *p* = 0.041). Likewise, none of the tibias reconstructed with locking plates failed cyclical load testing at 20,000 cycles, while 2/12 (17 %) of the tibias in the Steinmann pin group failed. Both failures were caused by cracks that began at the articular surface and caused exposure of the Steinmann pins at the proximal tibia articular surface. These results show the superiority of locking plate constructs to Steinmann pin constructs under the conditions tested.

Most studies evaluating defects left after curettage of giant cell tumors take place in the distal femur and proximal tibia, the two most common locations of these tumors [17]. Their propensity to form in the epiphyseal region of bone makes it difficult to reconstruct defects left by their removal [17]. Packing the defect with PMMA is a well-established technique used during these surgeries. The PMMA plays a structural role in supporting the articular surface [3]. The thermal necrosis caused by the cement as it hardens can play a role as an adjuvant treatment to decrease the rate of recurrence of the tumor [16]. The evidence for using Steinmann pin reconstruction with actual patient date is limited. Bini et al., in a retrospective review, published the results of 38 patients treated by a single senior surgeon with cement reconstruction and intramedullary Steinmann pin placement [15]; 84 % of patients had good or excellent results and there were no failures of the construct noted.

Some studies have questioned whether or not it is necessary to reconstruct distal femurs and tibias with PMMA and intramedullary Steinmann pins, or with PMMA alone. Murray et al. examined the effect of using intramedullary Steinmann pins to reconstruct distal femoral defects [11]. They found no difference in the stiffness, peak load to failure, and energy to failure between femurs treated with PMMA only, and femurs treated with PMMA and intramedullary Steinmann pins. In a similar study, Weiner et al. looked at the effect of intramedullary Steinmann pins to reconstruct proximal tibia defects [12]. This study also showed no difference in stiffness, peak load to failure, and energy to failure between tibias fixed with PMMA and PMMA with Steinmann pin constructs. Despite different biomechanics at the two locations tested, the inclusion of additional internal hardware did not seem to have any benefit. The authors of these studies also believed that the surgery to place the Steinmann pins is technically difficult, and not necessarily worth the extra effort to place them.

Other groups have shown a benefit to using Steinmann pin constructs to fix proximal tibia defects. Randall et al. looked at proximal tibia defects fixed with either PMMA alone or PMMA with intramedullary Steinmann pins [5]. They also examined the effect of placing different sized drill holes into the intact portion of the proximal tibia. The authors found the tibias in the small drill hole group (5-mm drill holes) had more cycles to failure and a greater load to failure in the groups with Steinmann pin reconstruction. They found no such difference between pin reconstruction and no-pin reconstruction when 10-mm drill holes were placed into the intact portion of the proximal tibia. They believed that the larger drill holes served to anchor the cement and increase the strength of the construct. However, when the drill holes were small, there was a significant benefit to having intramedullary Steinmann pins.

There is also an issue on how Steinmann pins can be used to reconstruct the defects left in proximal tibias. Toy et al. examined this by looking at three groups—proximal tibia defects reconstructed with cement alone, defects fixed with cement and intramedullary Steinmann pins, and defects fixed with cement and diverging Steinmann pins that engage the opposite, intact cortex in the proximal tibia [13]. They found the diverging pin construct to be mechanically superior to cement alone and cement with intramedullary Steinmann pins. They attributed this to the diverging screws being anchored into the strong bone of the intact tibial condyle. They also noticed less separation at the cement bone interface which they hypothesized was the cause of the decreased rate of intra-articular failure in this group. The tibias treated with a diverging screw pattern failed with extra-articular fractures. These fractures would be easier to treat in a patient and would not require a large endoprosthetic or allograft reconstruction because of destruction of the joint surface.

A previous study at our institution looked at giant cell tumor defects in distal femurs reconstructed with three different techniques—intramedullary Steinmann pins, crossed screws, and locking plate constructs [6]. The femora treated with locking plate constructs had a greater stiffness and load to failure than the other two groups. The locking plate group was 43 and 53 % stiffer than the intramedullary pin group and the crossed pin group, respectively. The failures noted in the locking plate group were extra-articular, which as previously stated by Toy et al., is desirable compared to intra-articular failures [13]. The locking plates were hypothesized to be stronger because they transferred the load from the joint surface to the intact femoral shaft more effectively than the other groups. The use of locking plates has been shown by other groups to be an effective treatment in other orthopedic oncology reconstructions [14].

There are several limitations to our study. As with any biomechanical study, the study conditions are idealized in a laboratory and might not adequately reflect physiological loading conditions. The forces we used attempted to simulate a patient walking upstairs/downstairs, which would put the patient’s proximal tibia under higher forces than just walking on level ground [7, 8]. Our applicability to other studies involving the proximal tibia might be limited because of the variability in creating proximal tibial defects. We chose to use lateral plateau defects for our study, and there is likely to be differences in loading between the medial and lateral tibial plateau. However, we only loaded the side of the tibia with the defect, and our results likely represent the direct strength on that side of the construct. There was variability in the size and bone quality of the tibias used in this study. We used paired tibias for the study in an attempt to negate this effect. The difference in size and bone quality of the tibias is also likely to represent the variety of patients that would be seen in clinical practice. We chose to use intramedullary Steinmann pins for our study, but other studies also look at using divergent pins anchored into the intact portion of the tibial plateau. We would have been unable to pair the tibias if we included a third experimental group into our study, so we chose not to include this group. Finally, the surgery to implant a locking plate would require a larger dissection and periosteal disruption that the surgery to implant intramedullary Steinmann pins. This could theoretically decrease the healing potential after surgery in these patients.

Our study showed a significant improvement in the stiffness and fatigue properties of proximal tibial defects treated with a locking plate construct compared to three intramedullary Steinmann pins placed in an inverted tripod position. None of the locking plate constructs failed during fatigue testing, while two of the other group failed with intra-articular fractures. The significant increase in fatigue properties likely translates into increased stability of the locking plate construct over time. We believe that proximal tibial locking plates represent a viable alternative treatment to the traditional treatment using Steinmann pin constructs.
